# Supplementary material for: The interactome of intact mitochondria by cross-linking mass spectrometry provides evidence for coexisting respiratory supercomplexes
Source: Mol Cell Proteomics. 2017 Dec 8;17(2):216–32. doi: 10.1074/mcp.RA117.000470 (PMC5795388; doi:10.1074/mcp.RA117.000470)
Supplement: Supplemental Data [file supp_RA117.000470_133922_0_supp_23978_fzffwf.zip › spectra_annotation/mito_DR_spectra_annotation/441-1-4-1-4-1.pdf]

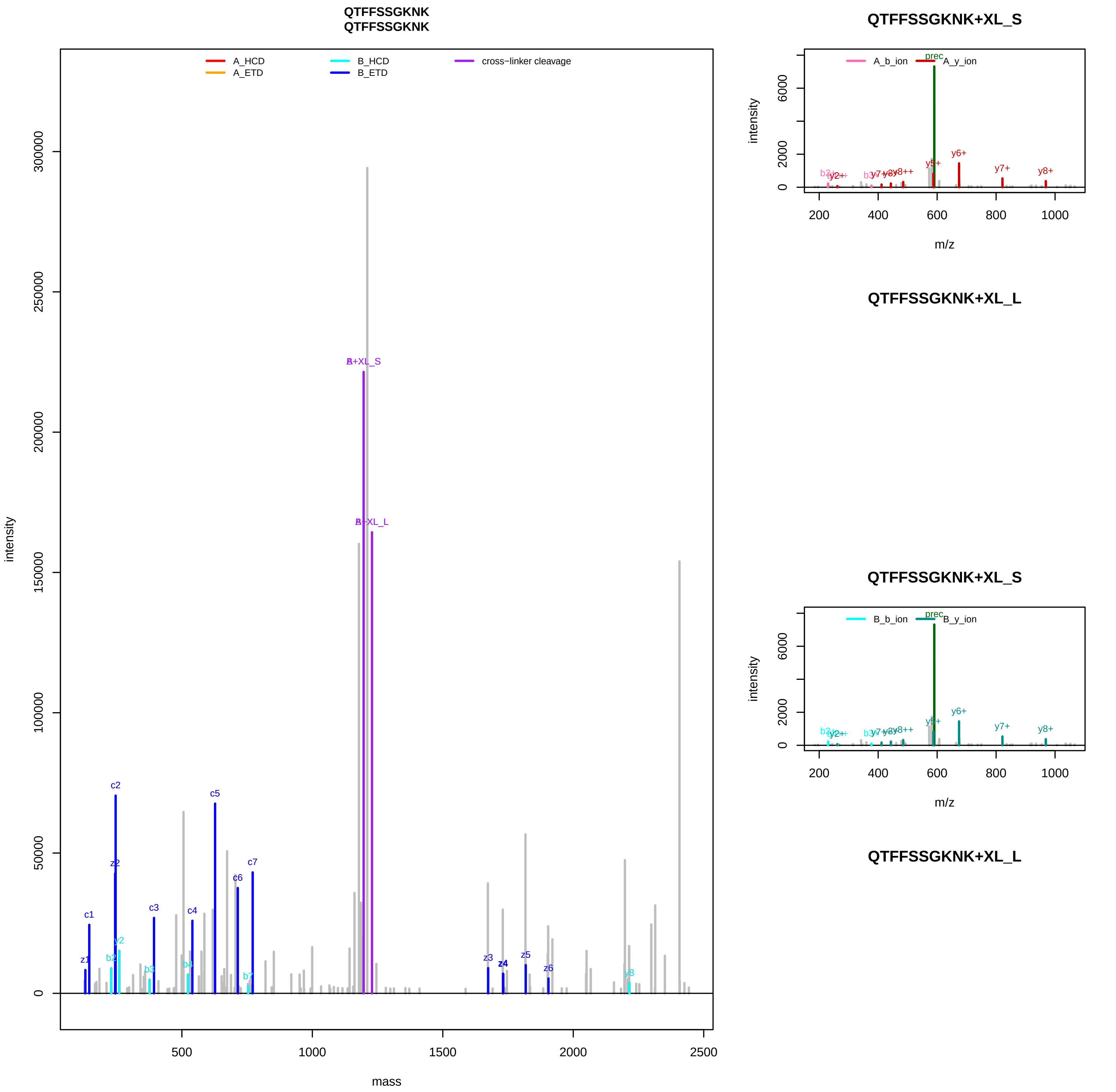
QTFFSSGKNK
QTFFSSGKNK
A_HCD
A_ETD
B_HCD
B_ETD
cross−linker cleavage
intensity
mass
B+XL_S
B+XL_L
c1
c2
z1
z2
b2
y2
c3
b3
c4
b4
c5
c6
c7
b7
z3
z4
z5
z6
y8
0
50000
100000
150000
200000
250000
300000
500
1000
1500
2000
2500
QTFFSSGKNK+XL_S
A_b_ion
A_y_ion
prec
y5+
y6+
y7+
y8+
m/z
0
2000
6000
200
400
600
800
1000
QTFFSSGKNK+XL_L
QTFFSSGKNK+XL_S
B_b_ion
B_y_ion
prec
y5+
y6+
y7+
y8+
m/z
QTFFSSGKNK+XL_L
